# Association Between *IL-28B* (rs8099917) and *IL-28B* (rs12979860) with Predisposition to Diseases Related to the HTLV-1: Systematic Review and Meta-Analysis

**DOI:** 10.3390/pathogens14050470

**Published:** 2025-05-13

**Authors:** Naomi Cuenca, Damarys Cordero, Brenda López-Ulloa

**Affiliations:** Grupo de Investigación en Aplicaciones Biotecnológicas (GIAB), Universidad Politécnica Salesiana (UPS), Carrera de Biotecnología, Campus María Auxiliadora, Kilómetro 19.5 Vía a La Costa, Guayaquil P.O. Box 09-01-2074, Ecuador; ncuencad@est.ups.edu.ec (N.C.); dcorderot2@est.ups.edu.ec (D.C.)

**Keywords:** IL-28B, HTLV-1, HAM/TSP, HTLV-1 related diseases

## Abstract

This research addresses *IL-28B* gene polymorphisms (rs12979860 and rs8099917) to determine their association with HTLV-1-related diseases; it aims to compare genotypic frequencies to identify predisposition or protection, considering population, disease, and controls. Given HTLV-1’s impact on immunity, this study seeks biomarkers for early diagnosis and intervention. A systematic search met inclusion criteria, such as open access bibliographic and experimental studies published in English between 2010 and 2024, and genetic factors linked to susceptibility to pathologies. Regarding exclusion criteria, bibliographic or experimental studies in organisms other than humans, unofficial sources, non-indexed journals, and scientific articles in languages other than English were ruled out. Statistical data analyses were assessed using meta-analysis, including forest plot and Q test of heterogeneity based on the I^2^ statistics. The analyzed data indicate associations between genotypes, such as CT, GG, CC, and TT of the rs12979890 and rs8099917 polymorphisms and the predisposition to various diseases, such as HCV, arthropathy, HAM/TSP, cytomegalovirus and Crimean–Congo hemorrhagic fever associated with HTLV-1; however, the observed inconsistencies, such as high heterogeneity, and deficiency of related information limit the consolidation of the findings. Further research is needed to clarify IL-28B genotype interactions and disease susceptibility in HTLV-1 infections.

## 1. Introduction

In 2012, the WHO released the latest global report, which estimates that about 10 million people were infected with human T-lymphotropic virus type 1 (HTLV-1), significantly affecting areas with deficiencies in the health system. However, it was not until 2018 that a group of experts and other stakeholders asked WHO to take action on HTLV-1, given increasing interest due to infection in some populations [1]. However, the number of infected people may be more significant because systematic epidemiology has not been carried out in some areas of endemic infection, and in 2020, a WHO technical report updated the data on HTLV-1 prevalence at the regional level, where it was highlighted that the country with the highest recorded prevalence of HTLV-1 in the general population is Australia, specifically among the Indigenous populations of the center of the country, where prevalences higher than 40% have been reported in older age groups [2,3].

HTLV-1 is a retrovirus that is transmitted through body fluids from people infected with the virus; these fluids include blood, breast milk, semen, and vaginal secretions [4]. The most common route of transmission is through sexual relations without a protective barrier, such as a condom. In contrast, transmission through contact with blood or tissues happens through blood or organ donation processes, in addition to sharing sharps such as needles, and vertical transmission from mother to child through breastfeeding [5].

HTLV-1 can cause T-cell leukemia in adults (ATL). However, only 2–5% of the infected people can develop it because of an imbalance in the host’s immune system, and ATL’s presence depends on the virus’s pathogenicity and the infected person’s immune system; the development of ATL also influences by the accumulation of genetic and epigenetic alterations in key genes related to host immunity, disrupting the immune balance, such as mutations in the T cell receptor pathway, nuclear factors, and DNA methylations [6,7,8,9]. Recently, the association of HTLV-1 with several chronic diseases has been determined. However, a direct association with chronic inflammatory diseases affecting the nervous system, such as myelopathy related to HTLV-1 or also called tropical spastic paraparesis (HAM/TSP), which progressively attacks the central nervous system and can trigger other diseases such as neurogenic bladder disease (NGBD) [10].

Predisposition to the mentioned diseases depends on the immunogenetic profile of the host since the interaction between the virus and the immune response plays a key role in the development [11]. Biomarkers such as cytokines and polymorphisms of their corresponding genes are considered to understand the immune response [12]. Some interleukins have been recognized, such as IL-10, IL-6, IL-17, IL-18, and IL-28B, which are factors that determine the prevalence of AMH or other infectious diseases such as hepatitis C (HCV), and chronic diseases such as arthropathy [13]. In particular, IL-28B, called interferon lambda 3 (IFN-λ3), plays a critical role in the innate immune response against viral infections [14]. This cytokine executes an antiviral activity by simulating interferon-simulated genes (ISGs), which limited the viral replication, so variations in the *IL-28B* gene, especially SNPs rs12979860 and rs8099917, have demonstrated influence in the expression level of this interleukin, relating genotype CC of rs12979860 and the TT genotype of rs8099917 with increased IFN-λ3 production, leading to a stronger antiviral response; on the other hand, unfavorable genotypes (such as TT for rs12979860) may result in weaker interferon responses, creating a permissive environment for viral persistence and immune evasion, which are critical aspects in HTLV-1 pathogenesis [15,16].

In the context of HTLV-1 infection, these polymorphisms could influence the clinical evolution of the disease by modulating the efficacy in the virus elimination, the level of chronic inflammation, and the immune control over the infected CD4+ cells, which are considered important aspects in the progression from the asymptomatic carrier state to severe conditions such as ATL or HAM/TSP [17]. Nonetheless, the information is poor, and there are still gaps in knowledge, so it is a field that needs further study.

Nevertheless, it has been possible to identify that the polymorphisms rs12979860 and rs8099917 of the *IL-28B* gene provide more information on the association between the mentioned diseases and HTLV-1 [18]. Therefore, by analyzing genotypic frequencies, the research investigates the association between *IL-28B* gene single nucleotide polymorphisms (rs12979860 and rs8099917) and HTLV-1-related diseases [19]. Given this background, we propose to determine whether these polymorphisms increase susceptibility to the disease or, on the contrary, provide a protective effect, considering the genotypic distribution, the characteristics of the population, the specific disease, and the control cases.

Although several studies have evaluated the association between the rs12979860 and rs8099917 polymorphisms of the *IL-28B* gene and the diseases related to the HTLV-1, the results have been contradictory, but this is because that the information is limited; some reports suggest a significant association with the development of HAM/TSP or ATL, while others have not found statistically relevant differences between the genotypes, the reason for this may be due to the type of population studied, besides depending on their genetic and immunological profile [6,13]. This absence of consensus in scientific literature highlights the need for a systematic analysis to integrate the available data and to determine these polymorphism roles more precisely in the pathogenesis of HTLV-1.

Since this virus confers risks such as the prevalence of sexually transmitted infections (STIs), ALT, HAM/TSP, cytomegalovirus, and leukemia through the alteration of the immune system, as well as keeping in mind that there are only treatments for the diseases associated to the virus, but there are no curative treatments for the HTLV-1 infection [1], the study seeks to provide information on the progression of the virus, its behavior according to the immunogenetic profile of the host, and the identification of possible biomarkers that allow the identification of a diagnosis and early intervention against chronic and infectious diseases related to HTLV-1.

## 2. Materials and Methods

### 2.1. Bibliographic Search Strategy

The literature search strategy employed in this systematic review focused on the guidelines issued by the PRISMA methodology [20]. Systematic reviews are classified as qualitative and quantitative; in this review, we incorporated quantitative analysis, which is referred to as meta-analyses, which focus on summarizing the results of numerous studies [21]. About the synthesis of data for the meta-analysis, dichotomous variables were applied; these included the number of cases, total cases, control number, and total control number in relation to the genotypic frequency (TT, CT, CC, and GG) of the rs8099917 and rs12979860 polymorphisms of the *IL-28B* gene linked to predisposition or protection to diseases associated with the HTLV-1 virus.

The methodological quality of the eleven studies included in the meta-analysis was assessed using the Newcastle Ottawa Scale (NOS), an observational cohort and case-control study tool [22]. The respective assessment was performed in three key domains: selection, which analyses the representativeness of the sample and the correct definition of the study groups; comparability, which determines the control of confounding factors by appropriate adjustments in the study design; and outcomes, which examine the quality of follow-up and measurement of events of interest. Scores were assigned to each study according to the fulfillment of the established criteria, with a maximum of nine indicating high methodological quality.

The systematic search covered a variety of databases, including PubMed, ScienceDirect, Springer, Web of Science, ProQuest, MDPI, and ProQuest, covering studies executed from 2010 to 2024. Regarding the formulation of search terms, the Boolean operators ‘AND’ were used to combine keywords such as HTLV-1, rs8099917 polymorphism, and rs12979860 polymorphism. The combinations of Boolean operators that proved suitable in academic search engines were ‘((HTLV-1) AND (polymorphism rs8099917))’ and ‘((HTLV-1) AND (polymorphism rs1297960))’.

The inclusion criteria are open-access bibliographic and experimental studies published in English to optimize data availability between 2010 and 2024, inclusive, and genetic factors related to the susceptibility to develop pathologies linked to the HTLV-1 retrovirus. About the exclusion criteria, bibliographic or experimental studies on organisms other than humans, studies evaluating polymorphisms other than rs8099917 and rs12979860 of the *IL-28B* gene associated with the HTLV-1 virus, non-official sources, and non-indexed journals and scientific articles in languages other than English were excluded.

### 2.2. Publication Bias

To visually assess the symmetry in the distribution of effect sizes of the included studies, a funnel plot was used. Asymmetry in this plot may indicate the presence of publication bias, which was statistically assessed using the Egger test [23]. Initially, the safety N was calculated using the Rosenthal method, which estimates the number of unpublished studies needed to invalidate the observed effect, thus allowing the potential impact of publication bias on the results to be assessed. In addition, the rank correlation test and the regression test, based on the standard error of the observed results, were applied to analyze the symmetric or asymmetric distribution of the funnel plot.

The Trim and Fill method was incorporated to complement the analysis and improve the estimation of the actual effect, allowing the detection and correction of potential asymmetries in the funnel plot. This approach helps estimate the number of missing studies due to publication bias and adjusts the data distribution to obtain a more accurate and balanced representation of the overall effect. Consequently, the application of these methods allowed a rigorous analysis of possible biases and variations in the data, strengthening the validity and credibility of the study’s conclusions regarding the association with the genotype frequency (TT, CT, CC, and GG) of the polymorphisms rs8099917 and rs12979860 of the *IL-28B* gene, linked to the predisposition to diseases associated with the HTLV-1 virus.

### 2.3. Statistical Analysis

For the statistical analysis of the data and obtaining the forest plot and funnel plot, the Rstudio software version 2022.02.0+443 was used; the odds ratio (OR) was analyzed as an outcome measure and evaluated using a random effects model because the studies used in this meta-analysis were carried out by different researchers, in addition to the heterogeneity rate. However, the random effects model was selected without considering heterogeneity [24]. The amount of heterogeneity, Tau^2^, was analyzed using the restricted maximum likelihood estimator. Likewise, the Q test for heterogeneity or Cochran’s test based on the Chi-square test and the I^2^ statistic was calculated, in turn, the data were clustered with 95% confidence intervals (95% CI) and a *p*-value greater than or less than 0.05 [25].

A *p*-value < 0.05 reflects statistically significant data. Data heterogeneity was represented by the symbol I^2^. An I^2^ value less than 25% represents low heterogeneity, while an I^2^ value between 25 and 50% reflects moderate heterogeneity, and an I^2^ value greater than 50% indicates high heterogeneity [26].

## 3. Results

### 3.1. Association Between IL-28B Gene and the Pathogenesis of HTLV-1

**Figure 1:**
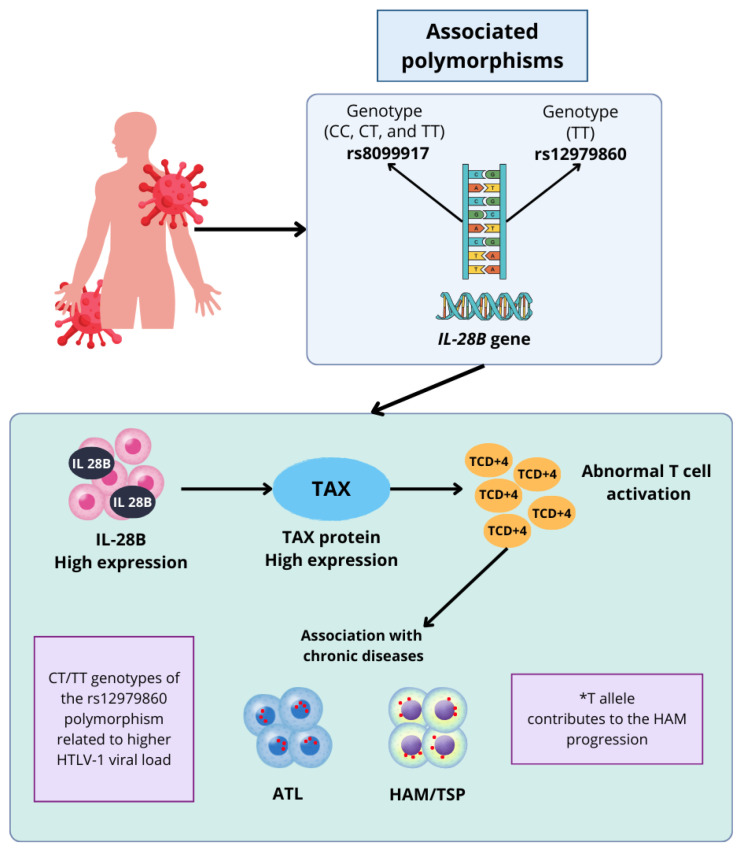
The role of *IL-28B* gene polymorphisms in HTLV-1 infection and associated diseases. *T refers to the T allele of the rs12979860 polymorphism of the *IL-28B* gene, specifically emphasizing this allele and not the complete genotype, such as CT or TT. Highlighting its possible relationship with the pathogenesis of the HTLV-1 infection and its influence on the development of associated diseases, the use of the asterisk indicates precisely that the individual effect of the T allele on the progression of the infection is discussed.

Figure 1 illustrates the association between the *IL-28B* gene polymorphisms and diseases related to HTLV-1 infection, highlighting their influence on viral progression and chronic disease development in the host. The rs8099917 and rs12979860 polymorphisms play a key role in the pathogenesis of ATL and HAM/TSP, as their genetic variations modulate the expression of IL-28B.

The rs8099917 and rs12979860 polymorphisms of the *IL-28B* gene play an important role in the pathogenesis of HAM/TSP; specifically, the TT genotype of the rs8099917 polymorphism is directly associated with an increased expression of the interleukin IL-28B, which causes an abnormal proliferation of CD4+ T lymphocytes, a mechanism that may promote chronic inflammation and increase the risk of development of mentioned diseases, which would result in the development of diseases such as ATL and myelopathy associated to HTLV-1. Regarding the rs12979860 polymorphism, the CC genotype confers a higher HTLV-1 viral load, suggesting that the *T allele is possibly closely related to the pathogenesis of the HTLV-1 infection, while contributing to both the development of AMH/TSP and its progression within the host infected by the virus.

Thus, the figure supports the arguments that *IL-28B* polymorphisms can act as immunogenetic markers that explain individual variability in disease development and provide insight into the host–pathogen interaction in HTLV-1 infection.

### 3.2. Study Characteristics

**Figure 2:**
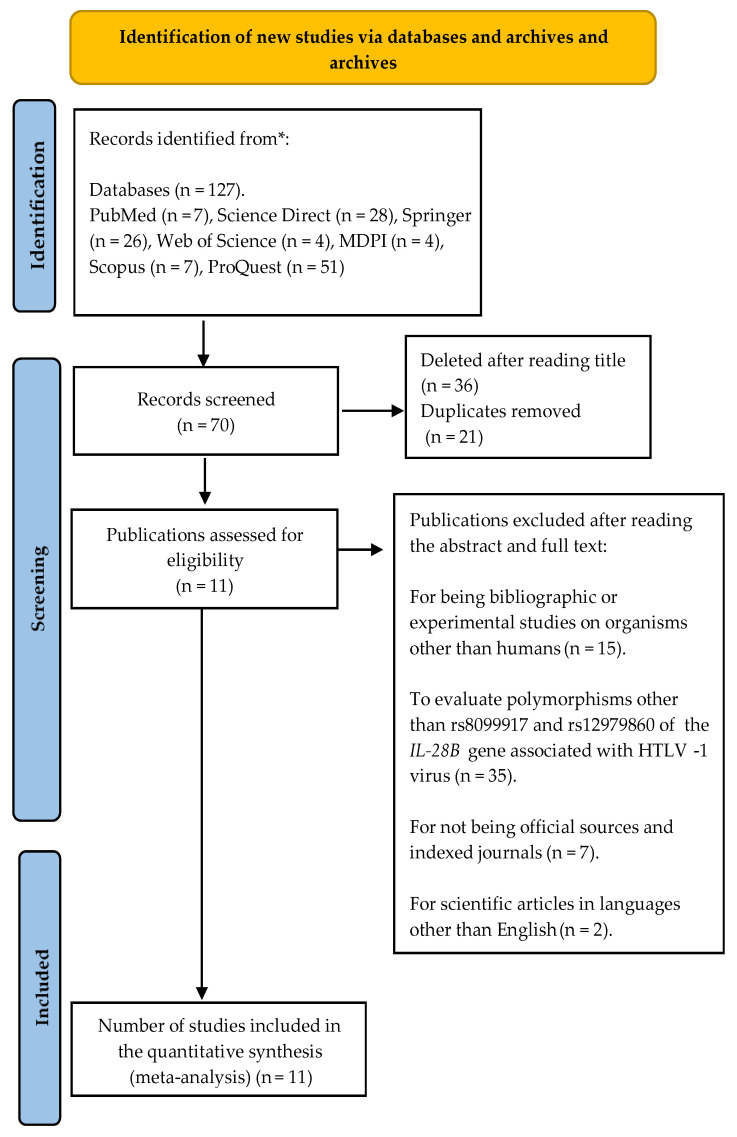
PRISMA 2020 flowchart for systematic reviews that included searches only in databases and registries. * Indicates that the identified records have been reported separately. Modified from [20].

A total of 127 results were identified and distributed, 7 were identified in PubMed, 28 in Science Direct, 26 in Springer, 4 in Web of Science, 4 in MDPI, 7 in Scopus, and 51 in ProQuest. According to the exclusion and inclusion criteria, and after the initial review based on the titles and eliminating duplicates, 70 articles were considered relevant. Subsequently, the abstracts and full texts were read, which led to the exclusion of 59 studies according to the criteria described in Figure 2. Finally, 11 articles that met the predefined inclusion criteria [19,27,28,29,30,31,32,33,34] were selected; these studies focused on the evaluation of the genotypic frequency of polymorphisms in relation to experimental cases and controls, in the context of the analysis of susceptibility to diseases associated with the HTLV-1 virus.

Table 1 shows the results of the quality assessment of the studies using the NOS, which indicate significant variations in the methodology of the included studies. The total score ranges from 5 to 9, reflecting differences in study selection, comparability, and outcome measurement. Most studies score high (8–9), indicating adequate methodological quality, with well-structured selection and good follow-up; however, some studies have lower scores (≤6), which may indicate risk of bias due to differences in participant selection or control of confounding factors of the studies selected for meta-analysis.

In the present meta-analyses, studies from diverse populations and diseases were included following rigorous selection criteria to ensure validity and comparability of results. Data integration was performed considering inclusion and exclusion criteria for the selection of studies sharing similar methodological characteristics, standardization of data by applying harmonization strategies to normalize the reported measures between studies, facilitating the comparison of results regardless of the different genotypic model, and type of pathology. Including diverse populations and diseases is based on biological and epidemiological evidence suggesting that the polymorphisms analyzed may be implicated in multiple medical conditions, facilitating a broader view of the genetic impact on different populations.

### 3.3. Interactions of the GG vs. TT Genotype Frequency of the IL-28B Gene rs8099917 Polymorphism

**Figure 3:**
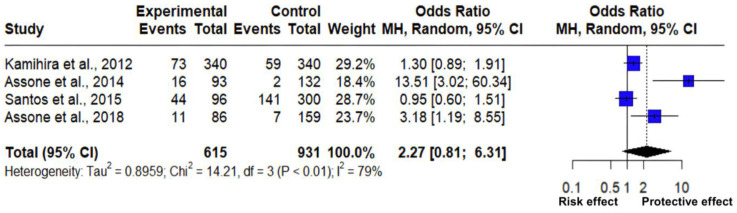
Forest plot of HTLV-1 associated with polymorphism rs8099917 of the *IL-28B* gene (GG vs. TT) [19,27,28,29].

The association between the frequency of the GG and TT genotypes of the rs8099917 polymorphism of the *IL-28B* gene was analyzed through a meta-analysis of four studies (Table 2), which included 1546 observations and 353 events. The estimated mean odds ratio based on the random effects model for this association was 2.267 (95% CI: 0.8145; 6.3060), which showed a statistically significant result (Z = 1.57, *p* = 0.1171) (Figure 3).

The analysis reflected an important and considerable heterogeneity between the association of the genotype frequency of the rs8099917 polymorphism and the predisposition to HTLV-1-related diseases, as indicated by different statistical measures: Tau^2^ = 0.8959 (ranging from 0.1267 to 19.2388), which is indicative of variance between the real effect sizes; Tau: 0.9465 (ranging from 0.3559 to 4.3862), indicating the standard deviation; I^2^ = 79% (ranging from 43.5% to 92.1%), reflecting a high percentage of variability due to differences between studies. On the other hand, H: 2.18 (ranging from 1.33 to 3.56), showing the association between the total variability and the variability evidenced within the study, and according to Cochran’s Q test: 14.21 with a *p*-value of less than 0.0026 (Table 3), which significantly supports the heterogeneity between studies. The high heterogeneity value shows that the studies employed varied significantly in their findings, suggesting that factors such as population characteristics, genetic background, age, and type of disease are associated with HTLV-1.

Regarding them, two of the studies reveal a clear relationship between the rs8099917 polymorphism and the predisposition to pathologies associated with HTLV-1, according to Figure 3, two studies [28,29] indicate that the GG genotype may present a different immune response against HTLV-1 infection, acting as a protective effect against HAM/TSP and hepatitis C virus in HTLV-1 carriers. On the other hand, a study [27] with an OR of 1.30 indicates a risk effect against HCV in the presence of the TT genotype; however, the square touches the no effect line, suggesting that there is a little significant relationship on the risk of suffering from arthropathy in HTLV-1 carriers. In addition, the final rhombus indicates the pooled effect size of all the data used, so its size represents the overall estimate of the combined effect of the four studies included in this meta-analysis, where its width reflects the confidence interval, demonstrating the precision of the estimate; the rhombus touches the no effect line, which means that there is a low significant association and greater variability in the results; however, it is possible to determine the existence of a susceptibility and protection effect for developing chronic diseases associated with HTLV-1 in the presence of the rs8099917 polymorphism and its genotypic frequencies, GG and TT.

### 3.4. Interactions of the CC vs. CT Genotype Frequency of the IL-28B Gene rs12979860 Polymorphism

**Figure 4:**
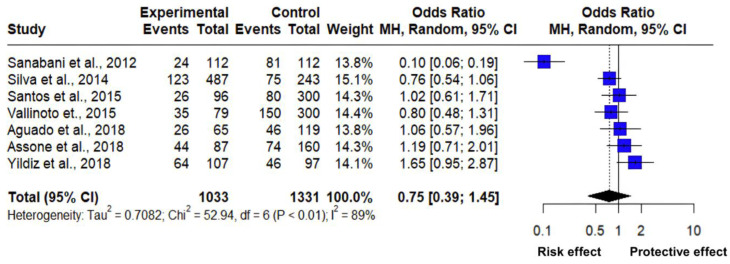
Forest plot of HTLV-1 associated with polymorphism rs12979860 of the *IL*-28B gene (CC vs. CT) [19,29,30,31,32,33,34].

The association between the CC and CT genotype frequency of the rs12979860 polymorphism of the *IL-28B* gene was analyzed through a meta-analysis of seven studies (Table 4). The estimated mean odds ratio based on the random effects model for this association was 0.7547 (95% CI: 0.3922; 1.4524). In addition, the Z value was −0.84 with a *p*-value = 0.3994, indicating that it does not differ significantly from zero and that there is no significant difference between the evaluated polymorphism and the susceptibility to suffering from pathologies related to HTLV-1 (Figure 4).

The heterogeneity in the analysis of the studies was significantly high with a Tau^2^: 0.7082 (range from 0.2482 to 3.8992); Tau: 0.8416 (range from 0.4982 to 1.9746); I2: 89% (range from 79.1% to 93.9%); H: 2.97 (varying from 2.19 to 4.03); and according to the Cochran’s Q test: 52.94 with a *p*-value less than 0.0001 (Table 5), which highlights the significant heterogeneity between the studies. Therefore, the high level of heterogeneity indicates significant variability, among the analyzed studies highlighting distinct differences.

The high heterogeneity observed (I^2^ = 79% and 89%) in the meta-analysis of the rs8099917 and rs12979860 polymorphisms indicates significant variability between the included studies. This suggests that the differences in effect sizes are not only due to random errors, but that there are underlying factors that are affecting the results, such as variability in the populations studied due to different genetic backgrounds, environmental factors, and even HTLV-1 prevalence, as well as different techniques used for the identification of polymorphisms and publication bias, since the preferential inclusion of studies with positive results may have an impact on the observed heterogeneity.

In this regard, a study [34] shows clear evidence that subjects with single nucleotide polymorphism rs12979860 of the CT genotype (Figure 4) may present a different immune response to HTLV-1 infection, with this genotype has a protective effect against Crimean–Congo hemorrhagic fever in subjects carrying HTLV-1. On the contrary, two other studies [19,31] with the presence of the CC genotype point to a risk effect against HCV and arthropathy. However, the square touches the line of no effect, suggesting that there is a low significant relationship between the risk of suffering from HCV, arthropathy, and cytomegalovirus in subjects carrying HTLV-1. However, one study [30] with the presence of the CT genotype clearly shows a risk effect against HAM/TSP disease in the presence of HTLV-1. Furthermore, the final diamond is on the side of the indicated risk effect; however, it touches the line of no effect, which means that there is a low significance association, but it can be determined that different diseases are associated with HTLV-1 but in the presence of the rs12979860 polymorphism and depending on the CC or TT genotype it can act as a risk effect and protect against the development of pathologies.

### 3.5. Result of the Publication Bias Analysis

**Figure 5:**
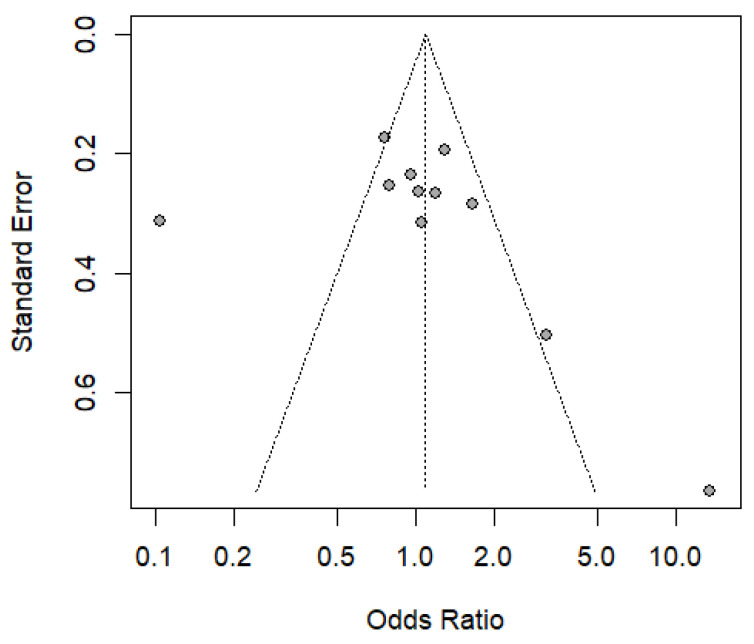
Funnel plot of chronic diseases associated with polymorphism rs8099917 and rs12979869 associated with the *IL-28B* with HTLV-1.

Figure 5 shows a funnel plot of the studies included in the meta-analysis, showing the existence of publication bias, due to its asymmetric distribution and significant differences. Concerning this, the regression test indicated the asymmetry of the funnel plot (*p* = 0.0202), but not the rank correlation test (*p* = 0.060), using the standard error of the observed results. The Trim and Fill method was applied to address this bias, which identified missing studies and adjusted the overall effect size by incorporating four additional studies. Following this correction, the recalculated random effects model yielded an odds ratio (OR) of 0.7225 [0.3936; 1.3263], with a *p*-value of 0.2943, indicating that the observed protective effect remains non-significant. Despite this adjustment, high heterogeneity (I^2^ = 89%) persists, suggesting considerable methodological and population differences across studies. Consequently, the results of the meta-analysis require further analysis and present a higher probability of overestimating the protective effect and the risk effect in relation to the genotype frequency of the HTLV-1-associated rs8099917 and rs12979869 polymorphisms.

## 4. Discussion

Different cytokines can significantly influence the reaction of the host immune system, as well as the different levels of HTLV-1 viral load, playing a direct role in the development of various pathologies, although a correlation between diseases such as uveitis and different polymorphisms has been identified, such as the polymorphism in the TNF type II gene with high levels of proviral load, which indicates that they could favor virus replication in individuals with HAM, the factors that cause HAM and the differences in viral loads between carriers with or without HTLV-1 symptoms are still not fully understood [13].

Identifying specific biomarkers would allow an early stratification of patients according to their susceptibility to develop HTLV-1-related pathologies. This genetic information could also be helpful in optimizing clinical follow-up strategies, particularly in endemic populations, and opening new lines in the research of targeted therapies [35]. Furthermore, using these biomarkers as prognostic tools could be a key to designing surveillance, prevention, and early intervention programs in at-risk individuals, thus improving the comprehensive management of chronic HTLV-1 infections.

Similarly, it was identified that different genotypic frequencies of polymorphisms are related to HTLV-1 infection. In one study [36], it was determined that the frequency of the −607 CC genotype of the *IL-18* gene was significantly lower in HTLV-1-infected individuals compared to uninfected individuals. In contrast, the −607 AC genotype of the same gene was found to be more common among HTLV-1 carriers [37]. In this context, the AA genotypic frequency of the −592 A/C polymorphism of the *IL-10* gene was found to be linked to an increased HTLV-1 proviral load, and the same polymorphism influences the decreased chances of developing HAM [38].

The analyzed data suggest associations between the CT, GG, CC, and TT genotypes of the rs12979890 and rs8099917 polymorphisms and predisposition to various diseases, including HCV, arthropathy, HAM/TSP, cytomegalovirus, and HTLV-1-associated Crimean–Congo hemorrhagic fever. However, the estimated OR of 0.75 [0.39; 1.45] indicates that, although there is a trend toward increased risk, the observed effect is not statistically significant since the confidence interval includes value 1. Furthermore, the high heterogeneity found 89% reflects considerable variability among the analyzed studies, attributed to differences in population characteristics, methodological approaches, and limitations in the available information.

On the other hand, IL-28B is an essential part of antiviral immunity through intracellular signaling and several antiviral factors activated in cells; although there are few studies on the association with HTLV-1, it has been demonstrated that polymorphisms in *IL-28B*, specifically, rs8099917 and rs12979860 are related to the progression of the infection and play a role in the origin of HAM. The association of these polymorphisms with HTLV-1 is determined by the CT/TT genotype with the highest proviral load and the highest expression of the *IL-28B* gene [39]. This scenario, since the mentioned genotype contributes to an anomalous activation and proliferation of CD4^+^ T lymphocytes, leads to favoring the development of an inflammatory process, and, since a higher pro-viral load is suggested, it would condition the existence of susceptibility to diseases associated with HTLV-1. Although there have been studies [40] that reveal whether *IL-28B* polymorphisms (rs8099917 and rs12979869) are associated with the risk of developing diseases in HTLV-1 carriers. Here, we investigate employing systematic review and meta-analysis, whether the genotypic frequency of these polymorphisms acts as a risk or protective factor for the susceptibility to develop diseases in HTLV-1 carrier subjects.

The present study reveals that the TT genotype of the rs8099917 polymorphism is associated with a higher expression of the *IL-28B* gene. In contrast, the GG genotype could confer protection against diseases such as HAM/TSP, because it is associated with lower levels of IL-28B. Based on this, and according to the meta-analysis of the GG vs. TT genotype of the rs8099917 polymorphism, three studies show OR > 1; one of them [27] presents an OR = 1.30 [0.89; 1.91], which suggests that the TT genotype is associated with a level of HTLV-1 infection co-infected with HCV in a population of Japanese origin. This result, compares with the study by [41], which, although not evaluated with HTLV-1, indicates that the genotype mentioned presents greater susceptibility to infection by the Hepatitis B virus in Egyptian patients. A comparison with a study evaluating the HTLV-1-associated TT genotype was not possible due to a lack of data.

Regarding the issue addressed, the odds ratio estimated at 2.27 [0.81; 6.31] suggests a protective effect on developing pathologies related to the HTLV-1 virus. However, given that the value 1, the observed effect does not present solid consistency; although there is a tendency towards lower risk, it is not possible to state with certainty this association, since the high heterogeneity (I^2^ = 79%) shows considerable variability among the studies analyzed, attributable to differences in population characteristics, methodologies used, deficiency of information, sample sizes, and the interaction of IL-28B with other genetic and environmental factors. These findings agree with what has been reported [27], where it was observed that there are few studies focused on the analysis of the association of this polymorphism in the *IL-28B* gene with HTLV-1 infection, but identified that it is widely linked to the progression of infection by this retrovirus.

As for the genotypic interactions between CC and CT, the results of this study indicate that the CT genotype of the rs12979860 polymorphism is associated with an increased risk of developing diseases in the presence of HTLV-1, while the CC genotype could act as a protective factor against pathologies such as HCV. According to the meta-analysis carried out, it was identified that three studies show OR < 1, of which two are related to the CC genotype. For example, the study [31] reports an OR of 0.76 [0.54; 1.06], suggesting that the CC genotype may play a protective role against HCV infection, possibly reducing susceptibility to this disease or showing a non-significant effect in this specific case. This finding is contrasted with the study by [42], which analyzed the absence of the mentioned polymorphism in relation to the neurological status in Afro-Caribbean individuals infected with HTLV-1, indicating that the mentioned genotype does not present a significant association with neurological phenotypes associated with HTLV-1.

Similarly, four studies report an OR higher than 1; one of them [33] shows an OR of 1.06 [0.57; 1.96], indicating that the CT genotype in the Spanish population could be linked to a higher susceptibility to cytomegalovirus. This result contrasts with the study by [43] that describes a higher prevalence of the CT genotype of the rs12979860 polymorphism in patients with HAM/TSP associated with HTLV-1. Consequently, it is inferred that the presence of the T allele could play a role in the infection’s development and in the disease’s development, and in the disease’s clinical outcomes, favoring the development of HAM.

In the analyzed context, the estimated OR of 0.75 [0.39; 1.45] indicates a possible increased predisposition to pathologies related to the HTLV-1 virus. However, because the confidence interval includes value 1, the observed effect cannot be considered statistically significant; although there is a trend toward greater risk, it is not feasible to confirm a strong association. Furthermore, the high level of heterogeneity (89%) highlights considerable variability among the reviewed studies, attributable to divergences in the characteristics present in the populations, methodological approaches, and limitations in the available information. These results agree with what has been described [30], who pointed out that neither mutations of the homozygous TT genotype nor those of the heterozygous CT, nor combinations between these genotypes, are associated with an increased risk of developing myelopathy/tropical spastic paraparesis in the Brazilian population. This analysis highlights the complexity of the genetic and environmental factors involved in the pathogenesis of diseases.

According to previous studies, no clear relationship has been identified between the TT and CT genotypes or combinations of them, and the risk of developing myelopathy/tropical spastic paraparesis in the Brazilian population. Therefore, the interaction between genetic and environmental factors in the pathogenesis of these diseases is complex and requires further research using comprehensive approaches that examine the interaction of *IL-28B* gene polymorphisms and susceptibility to developing these diseases or chronic conditions in the presence of HTLV-1 infection.

The high heterogeneity observed in the meta-analysis (I^2^ = 79% and 89%) indicates significant variability between studies, attributable to differences in population, methodological, and pathological characteristics. Factors such as genetic diversity, the use of different genotyping techniques, and variability in the clinical criteria employed may affect the results’ comparability. Furthermore, the funnel plot’s asymmetry suggests publication bias, reflecting a possible overestimation of the protective effect or risk associated with the rs8099917 and rs12979860 polymorphisms.

The recording of the high heterogeneity value was due to the main factor, the limited information; even though these *IL-28B* gene polymorphisms have been related to the HTLV-1, the information is scarce, which made it challenging to obtain the data. In addition, many articles did not have case controls and case studies, variables that are indispensable to perform the meta-analysis, which limited, even more, the selection of the corresponding literature. Above all, the variation in the number of the population evaluated from one study to another was another challenge to be tackled during the development of our study. It is highlighted that the information collected complied with the established year range for the search.

Subgroup analyses, meta-regression, and exclusion strategies for studies with low methodological quality are recommended to address this heterogeneity. The application of the Trim and Fill method, which incorporated four additional studies, partially corrected the publication bias, although the variability remains considerable. These findings highlight the need for future studies with more standardized approaches to improve the precision in estimating the relationship between *IL-28B* gene polymorphisms and susceptibility to HTLV-1-related diseases.

The findings of this study suggest the need for future research with multicenter designs and more diverse populations, which would allow for the reduction of the observed heterogeneity to improve the validity of the results. It would be beneficial to learn more about the interaction of polymorphisms with other genetic factors; while information exists on other markers, it is minimal when it comes to the *IL-28B* gene. Therefore, understanding environmental factors would also allow for the development of predictive models that facilitate the identification of the clinical risk of certain HTLV-1 carriers and their predisposition to develop conditions of scientific and clinical interest.

Recognizing these genetic markers as a guide for understanding HTLV-1 would allow healthcare professionals to make more informed decisions regarding differentiated clinical follow-up, intensifying surveillance in patients with higher-risk genotypes as specified throughout the study. Furthermore, integrating diagnostic protocols could improve prognosis through timely preventive interventions, thus becoming a basis for developing clinical risk profiles and opening new avenues for researching targeted therapies that modulate IL-28B expression or its signaling pathway.

## Figures and Tables

**Table 1:** Quality assessment of studies using the Newcastle Ottawa Scale.

Study	Selection	Comparability	Results	Total
Kamihira et al., 2012 [27]	3	2	3	8
Assone et al., 2014 [28]	3	1	2	6
Santos et al., 2015 [19]	4	2	3	9
Assone et al., 2018 [29]	2	1	2	5
Sanabani et al., 2012 [30]	4	2	3	9
Silva et al., 2014 [31]	4	2	3	9
Vallinoto et al., 2015 [32]	4	2	3	9
Aguado et al., 2018 [33]	3	1	2	6
Yildiz et al., 2018 [34]	3	2	3	8

**Table 2:** Distribution of GG vs. TT genotypic frequency of the rs8099917 polymorphism of the *IL*-*28B* gene.

Pathology	Country	Genetic Model	Cases (n)	Total (n)	Control (n)	Total (n)	Reference
HCV	Japan	TT	73	340	59	340	[27]
HAM/TSP	Brazil	GG	16	93	2	132	[28]
Arthropathy	Brazil	TT	44	96	141	300	[19]
HAM/TSP	Brazil	GG	11	86	7	159	[29]

**Table 3:** Quantification and test of heterogeneity in relation to genotypic frequency (GG vs. TT).

Z	Tau	Tau^2^	I^2^	H	Q	*p*
1.57	0.947	0.8959	79%	2.18	14.209	0.003

**Table 4:** Distribution of CC vs. CT genotypic frequency of the rs12979860 polymorphism of the *IL*-*28B* gene.

Pathology	Country	Genetic Model	Cases (n)	Total (n)	Control (n)	Total (n)	Reference
HAM/TSP	Brazil	CT	24	112	81	112	[30]
HCV	Brazil	CC	123	487	75	243	[31]
Arthropathy	Brazil	CC	26	96	80	300	[19]
HAM/TSP	Brazil	CT	35	79	150	300	[32]
Cytomegalovirus	Spain	CT	26	65	46	119	[33]
HAM/TSP	Brazil	CT	44	87	74	160	[29]
Crimean–Congo hemorrhagic fever	Turkey	CT	64	107	46	97	[34]

**Table 5:** Quantification and test of heterogeneity in relation to genotypic frequency (CC vs. CT).

Z	Tau	Tau^2^	I^2^	H	Q	*p*
−0.843	0.842	0.7082	88.7%	2.97	52.94	0.0001
